# The Etiology of Bloodstream Infections at an Italian Pediatric Tertiary Care Hospital: A 17-Year-Long Series

**DOI:** 10.3390/pathogens13080675

**Published:** 2024-08-09

**Authors:** Chiara Russo, Marcello Mariani, Martina Bavastro, Alessio Mesini, Carolina Saffioti, Erica Ricci, Elisabetta Ugolotti, Roberto Bandettini, Elio Castagnola

**Affiliations:** 1 Department of Neuroscience, Rehabilitation, Ophthalmology, Genetics and Maternal and Child Sciences (DiNOGMI), University of Genoa, 16132 Genoa, Italy; 2 Infectious Diseases Unit, Department of Pediatrics, IRCCS Istituto Giannina Gaslini, 16147 Genoa, Italy; 3 Division of Infectious Diseases, Department of Health Sciences (DISSAL), University of Genoa, 16132 Genoa, Italy; 4 Infectious Diseases Unit, IRCCS Ospedale Policlinico San Martino, 16147 Genoa, Italy; 5 Laboratory of Microbiology, IRCCS Istituto Giannina Gaslini, 16147 Genoa, Italyrobertobandettini@gaslini.org (R.B.)

**Keywords:** bloodstream infections, pediatrics, epidemiology, antibiotic resistance

## Abstract

Knowledge of epidemiology is essential for guiding correct antibiotic prescription, reducing bacteremia-associated mortality, and implementing targeted infection control programs. However, only a few studies have reported on the epidemiology of bloodstream infections (BSIs) in pediatrics. We performed a retrospective analysis of all BSIs (excluding those caused by common skin contaminants) diagnosed from 2006 to 2022 in patients younger than 18 years who were treated at an Italian pediatric tertiary care hospital. Overall, 2395 BSIs were recorded, including 2207 (92.15%) due to bacteria and 188 (7.85%) due to fungi. The incidence rate (BSIs/10,000 hospital discharges, IR) of bacterial BSIs significantly increased during the study period. In particular, BSIs caused by *S. aureus* (including MRSA), *Enterobacterales* (including ESBL and AmpC producers), *Enterococcus* spp., and *P. aeruginosa* became more common. The frequency of carbapenem-resistant strains was <1% and stable over time. Conversely, there was a significant reduction in the incidence of BSIs due to *S. pneumoniae.* The BSIs were stratified by patient age, and *S. aureus* was the most frequent cause of BSIs in all age groups, while *E. coli* was the most frequent in the *Enterobacterales* family. *S. agalactiae* was the third most frequent cause of neonatal early-onset BSIs. The prevalence of *Enterococcus* spp. increased in the subgroups from 8 days to 5 years of age, while *P. aeruginosa* became more prevalent in children over 5 years of age. *S. aureus* was also the most frequent isolate in both community- and hospital-onset BSIs, followed by *E. coli*. The prevalence of multidrug-resistant (MDR) pathogens was very low. It was <5% for both Gram-positive (i.e., MRSA and VRE) and Gram-negative (ESBL, AmpC, and carbapenem-resistant) pathogens, and MDR pathogens were almost exclusively detected in hospital-onset BSIs. Fungi accounted for just under 8% of BSIs. *C. albicans* was the most frequently isolated strain, followed by *C. parapsilosis*. Notably, the IR of fungemia did not change significantly during the study period, in spite of an increase in the absolute number of events. The continuous monitoring of local epidemiology is essential to identify changes in the IRs of pathogens and antibiotic susceptibility and to guide antibiotic treatments, especially in the phase when antibiograms are not yet available.

## 1. Introduction

Bloodstream infections (BSIs) significantly contribute to pediatric morbidity and mortality, particularly when they trigger sepsis and septic shock. In 2017, approximately 25 million children worldwide developed sepsis, resulting in over 3 million deaths [1]. In children, guidelines recommend starting antimicrobial therapy within 3 h after the diagnosis of sepsis, and even within 1 h in cases of septic shock [2], since the prompt and appropriate treatment of BSIs is crucial to prevent progression to septic shock and death. However, the identification of the causative agent of a BSI and the determination of its antibiotic susceptibility may take 48–72 h, thus making it necessary to carry out empirical antimicrobial therapy based on local epidemiology, also considering factors such as age, sex, and the presence of comorbidities [2]. In the era of antimicrobial resistance (AMR) [3,4], knowledge of local epidemiology is essential for guiding empirical therapy, especially in critically ill patients who may require broad-spectrum coverage that includes multidrug-resistant (MDR) organisms [5,6]. AMR prevalence varies over time, in different geographical regions, and even among units within the same hospital [7]. General epidemiology and AMR rates can change over time due to the introduction of vaccines, peripartum/surgical prophylaxis, pandemics (e.g., SARS-CoV-2), antibiotic prescription practices, and infection control measures [8]. Despite the recognized importance of epidemiology in guiding antibiotic prescription [9], few studies have reported on the epidemiology of BSIs in pediatric settings [10,11,12].

This study aims to describe the etiology of BSIs in children hospitalized at a tertiary care pediatric hospital in northern Italy, focusing on the age distribution, community- or hospital-onset, and major susceptibility patterns, while also analyzing epidemiological variations over time.

## 2. Materials and Methods

This study presents a retrospective review of all positive blood cultures collected from children admitted to the IRCCS Istituto Giannina Gaslini (IGG), Genoa, Italy, from 1 January 2006 to 31 December 2022. The IGG is a tertiary care pediatric hospital in northern Italy that serves as a local pediatric hospital and a referral hospital nationwide and for many foreign countries.

BSIs diagnosed at admission or during hospitalization in patients aged 0–18 years were included in this study. Episodes were identified in a microbiology database. The only demographic variable, collected anonymously, was the age at BSI diagnosis. Additionally, the ward where blood cultures were sampled was recorded. A BSI was defined by the isolation of a pathogenic microorganism from at least one aerobic/anaerobic set of blood culture bottles. For bacterial BSIs (bacteremias), blood cultures growing common skin contaminants [13] were excluded from the analysis. According to the literature, contaminants were defined as microorganisms that are thought to be introduced to cultures during specimen collection or processing and are not generally pathogenic for patients [14]. Microorganisms with contamination rates ≥ 50% were considered contaminants [14,15,16,17] (see Supplementary Table S1). Blood cultures positive for fungi (fungemias) were included. The patients could have repeated positive blood cultures: cultures from an individual patient that were positive for the same pathogens within two weeks of the initial isolate were considered a single incident.

Among the bacteremias, methicillin-resistant *Staphylococcus aureus* (MRSA) were defined as *S. aureus* resistant to oxacillin, and vancomycin-resistant *Enterococcus* spp. (VRE) were defined as *Enterococcus* spp. resistant to vancomycin. Among the Gram-negative pathogens, due to the occasional unavailability of molecular tests to differentiate between extended-spectrum *β*-lactamases (ESBLs) and AmpC throughout the study period, ESBL-producing microorganisms were pragmatically defined as those resistant to third-generation cephalosporins (cefotaxime and/or ceftazidime) and a fourth-generation cephalosporin (cefepime) (third/fourth generation cephalosporins, 3/4GC). AmpC-producing microorganisms were defined as those resistant to a third-generation cephalosporins but susceptible to cefepime. Carbapenem resistance was defined as resistance to meropenem. Resistance to specific antibiotics was defined based on the European Committee on Antimicrobial Susceptibility Testing (EUCAST) guidelines, which classify microorganisms as follows: susceptible (S), when there is a high likelihood of therapeutic success using a standard dosing regimen; susceptible increased exposure (I), when there is a likelihood of therapeutic success if exposure to the agent is increased by adjusting the dosing regimen or by its concentration at the site of infection; resistant (R), when there is a high likelihood of therapeutic failure even with increased exposure [18]. The category “I” was combined with “S” since the hospital policy typically administers the maximal doses of antibiotics. Interpretations were used instead of the minimal inhibitory concentration (MIC) values due to the potential changes in the EUCAST interpretation of MIC over the years.

BSIs were classified based on age, considering neonatal BSI for under 30 days, and early-onset BSI for those ≤7 days of age [12]. Other age groups included infants (1–3 months), older infants (3 months–1 year), toddlers (1–5 years), and children (>5 years) [13].

A BSI was categorized as community-onset if the positive blood culture was performed in the emergency or short-stay observation ward; it was categorized as hospital-onset if the blood culture was collected in any other hospital wards.

### Statistical Analysis

A descriptive analysis was conducted for the distribution of different pathogens across the entire patient population, stratified by age group and the ward where the blood culture was sampled. Descriptive analyses were used to explore the demographic factors of patients with BSIs (i.e., age and ward at the time of the BSI) and to summarize the bacterial isolates and anti-microbial susceptibility patterns. The annual incidence rate (IR) of BSIs was calculated as the ratio between the number of BSIs diagnosed over one year and the number of hospital discharges in that year, normalized to 10,000 discharges. The Spearman test was used to evaluate changes in the IR during the study period (Social Science statistics https://www.socscistatistics.com/). Two-sided *p* values < 0.05 were considered statistically significant.

## 3. Results

During the study period, a total of 2395 BSIs were diagnosed: 2207 (92.15%) bacteremias and 188 (7.85%) fungemias. Among the bacteremias, 47.34% were caused by Gram-negatives and 44,81% by Gram-positives. Fungi accounted for 7.85% of the isolates. The mean ages at the time of a BSI was 52.87 months (range: 0.01–215.37) for bacteremias and 48.36 months (range: 0–216.27) for fungemias.

### 3.1. Pathogen Distribution

Specific data on the prevalence of different etiologies during the study period are reported in Table 1 and Table 2. ESKAPE pathogens (*Enterococcus faecium*, *Staphylococcus aureus*, *Klebsiella pneumoniae*, *Acinetobacter baumannii*, *Pseudomonas aeruginosa*, and *Enterobacter* spp.) accounted for 44.7% of episodes (*n* = 1070). *Enterobacterales* were the most frequently isolated, representing 31.03% of all BSIs, with *E. coli* being the most representative of the group, isolated in 11.27% of all BSIs. Among the *Enterobacterales*, the proportion of ESBL and AmpC producers was <5%, and only 10 isolates (0.42%) were resistant to meropenem. Among the non-fermenting Gram-negative pathogens, *P. aeruginosa* was the most commonly isolated (*n* = 132, 5.51%), and rarely resistant to 3/4GC, piperacillin/tazobactam, or meropenem (<1% of cases). Among the Gram-positive pathogens, *S. aureus* was the most representative isolate, and the most frequent cause of BSIs (*n* = 497, 20.00%), with 97 strains identified as MRSA (4.05% of all BSIs, but 20.25% of all *S. aureus* strains). *Enterococcus* spp. were the second most representative Gram-positive pathogen (*n* = 310, 12.94%), but VRE were isolated in <1% of cases. Notably, *S. pyogenes* was isolated only in 0.8% (20/2395) of BSIs. Among the fungemias, *Candida albicans* and *C. parapsilosis* were the two most frequently isolated yeasts (*n* = 71 (1.96%) and *n* = 59 (2.46%), respectively).

### 3.2. Age Group Stratification

Table 3 reports the frequencies of the most representative microorganisms stratified by age group. *S. aureus* was among the top three pathogens in all age subgroups, while *S. agalactiae* was the third most frequent cause of neonatal early-onset BSI. Among the Gram-negative pathogens, *E. coli* was the most representative pathogen across all the age subgroups. The prevalence of *Enterococcus* spp. increased in neonates and infants, while *P. aeruginosa* became more prevalent in children. *C. albicans* was mainly detected in the first month of life, while C. parapsilosis was more frequently isolated in older patients.

### 3.3. Community- vs. Hospital-Onset Infections

Table 4 reports the etiologies of BSIs classified as community- or hospital-onset. In the community-onset infections, *S. aureus* was the most frequently isolated pathogen, followed by *E. coli*, *S. pneumoniae*, and *S. agalactiae.* Out of the hospital-onset infections, *S. aureus* was again the most frequent isolate, followed by *E. coli*, *E. faecalis*, and *K. pneumoniae*. Interestingly, the proportions of *S. pyogenes* BSIs were quite similar in the community- and hospital-onset infections (60% (*n* = 12/20) and 40% (*n* = 8/20), respectively). Notably, only a few MRSA—(6/97 and 6.18%), ESBL—(2/94 and 2.13), and AmpC—(2/67, 2.98%) producing *Enterobacterales* were isolated in the community-onset BSIs. Fungemia was nearly exclusively diagnosed in the hospital-onset infections, with *Candida albicans* representing about 3% of all the identified causes of BSI.

### 3.4. Incidence Rates and Trends over Time

**Figure 1:**
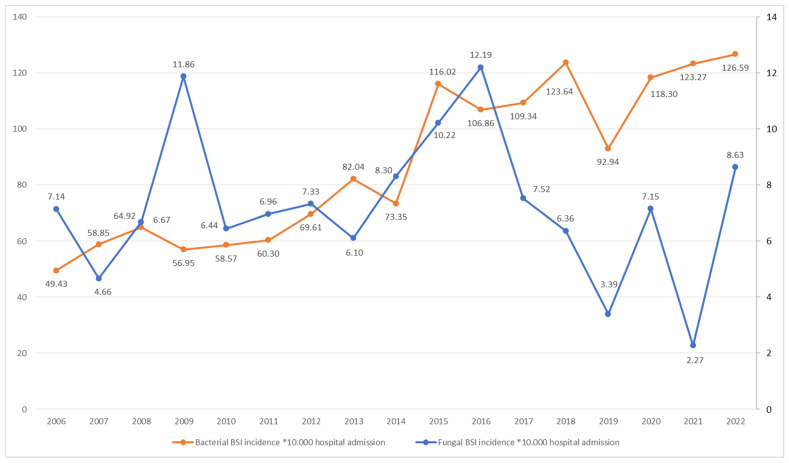
Incidence rate of bacteremias and fungemias by year of observation. Legend: BSI, bloodstream.

Figure 1 illustrates the overall IRs of bacteremias and fungemias and their trends over time. A statistically significant increase in the bacteremia IR was observed (rs = 0.85454, *p* = 0.00081), whereas no significant change was observed for fungemias (rs = 0.01226, *p* = 0.96277). Table 5 reports the IRs of different causes of BSIs over the years. Statistically significant increases were found in the BSIs due to *S. aureus* (including MRSA), *Enterobacterales* (including ESBL and AmpC producers), *Enterococcus* spp., and *P. aeruginosa*. In contrast, there was no significant change in the IRs of VRE, meropenem-resistant *Enterobacterales*, or *P. aeruginosa* resistant to 3/4GC, piperacillin/tazobactam, or meropenem. Additionally, statistically significant reductions were observed in the IRs of BSIs due to *S. pneumoniae*.

## 4. Discussion

In this study, we have reported the epidemiology of BSIs diagnosed at a pediatric Italian tertiary care center from 2006 to 2022. Despite being based on data from a single center, the scarcity of pediatric data on BSI etiology and the long time span covered by this study make these results important.

Unlike other studies that have reported stable BSI hospitalization rates [19], we observed a significant increase in the bacteremia IR, while the increase in the absolute number of fungemias did not result in a significant change in the IR. *Enterobacterales* was the most frequently identified pathogen family, with *E. coli* being the most prevalent. *S. aureus*., which was the most frequent isolate in absolute terms, showed an increasing IR over the years, along with *Enterococcus* spp., *S. agalactiae*, *Enterobacterales*, and *P. aeruginosa*. Other authors reported different trends for *Enterococcus* spp. and *S.pyogenes* [19]. Notably, in our series, *S.pyogenes* only represented less than 1% of all BSI cases. The low IR of BSIs due to *H. influenzae*, *S. pneumoniae*, and *N. meningitidis* likely reflects the impact of vaccines introduced in Italy between 1995 and 2001 [20,21,22,23]. While the IR of BSIs due to *H. influenzae* and *N. meningitidis* remained low and stable, the IR of BSIs due to *S. pneumoniae* significantly decreased. This reduction, in a region with a vaccination rate of >90% [24], is likely due to the introduction of the 13-valend pneumococcal-conjugate vaccine (PCV13), which started to substitute the PCV7 vaccine in 2010 [19].

Stratification by age showed that *S. aureus* was the most frequent cause of BSIs across all age groups, followed by *E. coli. S. agalactiae* was significant in neonates aged ≤ 7 days, as previously observed [25,26], while *E. faecalis* and *P. aeruginosa* were more prevalent in older children. Other studies have indicated a higher prevalence of *S. pneumoniae* and *Salmonella* spp. [12], contrasting with our findings of a higher prevalence of *E. faecalis* and *P. aeruginosa.* Fungi were a rare cause of BSI, accounting for just under 8%, with *C. albicans* and *C. parapsilosis* being the two most represented yeasts. This low prevalence aligns with other recent reports [27]. An important limitation of our study is the exclusion of coagulase-negative staphylococci (CoNS) from the analysis. CoNS are often considered pathogens causing late-onset sepsis in low-birth weight infants, a population where contaminants could play a significant role in the epidemiology. In other age groups, CoNS may be considered pathogens in cases of central line-associated bloodstream infection (CLABSI). However, since other diagnoses were not included and this was a laboratory-based study, it is impossible to know if a central line was present at the time of blood culture. Since CLABSIs were not the main focus of this study and the presence of the line could not be determined, it is not possible to ascertain if isolates considered contaminants could be true pathogens.

When comparing community- vs. hospital-onset BSIs, *S. aureus* was more frequent in community-onset infections, followed by *E. coli*. In this setting, *S. pneumoniae* and *S. agalactiae* were also common. In contrast, in hospital-onset BSIs, *S. aureus* and *E. coli* remained frequent, with a significant presence of *E. faecalis* and *K. pneumoniae*. Fungemia was found almost exclusively in the hospital-onset BSIs.

Finally, regarding AMR pathogens, ESKAPE pathogens accounted for 20% of the BSIs, consistent with other studies [28]. MRSA represented nearly 20% of the *S. aureus* strains, but its proportion remained < 10% across all age groups, with a slight prevalence in the hospital-onset BSIs. VRE were rare, while ESBL- and AmpC-producing *Enterobacterales* showed increasing trends. No significant changes were observed in carbapenemase-producing *Enterobacterales* or MDR *P. aeruginosa*. The low prevalence of MDR pathogens at our institute, which differs from observations at other centers and throughout Italy [29,30], could be attributed to the strict screening and isolation protocols adopted by our hospital [31]. However, it is important to note that the data from 2020–2022 could have been influenced by the widespread reduction in MDR hospital pathogens that occurred during the SARS-CoV-2 pandemic [32]. Additionally, we did not study the epidemiology in 2023, when there might have been an increase in MDR microorganisms due to increased antibiotic prescription during the pandemic [33,34]. We also identified an increase in *S. aureus* MIC for vancomycin [35], and a concerning number of cefiderocol-resistant Gram-negative pathogens [36]. To further underscore the association between MDR pathogens and hospitalization/antibiotic exposure, we observed that almost all MRSA, VRE, MDR *Enterobacterales*, and *P. aeruginosa* were responsible for hospital-onset BSIs, while only a few MRSA-, ESBL-, and AmpC-producing *Enterobacterales* were isolated in the community-onset BSIs.

In conclusion, understanding local epidemiology is essential for guiding empirical antibiotic treatment, as the incidence of causative pathogens can change over time and among geographical areas. Despite the low proportion of MDR pathogens identified in our series, the high percentage of ESKAPE pathogens and the increases in the IRs of some resistant pathogens underscore the necessity for continued screening and infection control measures, along with prudent use of antibiotics and accurate and up-to-date diagnostic testing.

## Figures and Tables

**Table 1:** Absolute numbers and proportions of different pathogens causing bloodstream infections during the study period.

Causative Pathogen of BSI	Absolute Number, *n*	Percentage of Total BSI, %
*S. aureus*	479	20.00
*S. pyogenes*	20	0.84
*S. pneumoniae*	38	1.59
*S. agalactiae*	65	2.71
Viridans *Streptococcus* spp.	140	5.85
*Lactobacillus*	8	0.33
*Enterococcus* spp.	310	12.94
*E. faecalis*	214	8.94
*E. faecium*	75	3.13
*Listeria*	8	0.33
Other Gram-positive	3	0.13
Gram-positive anaerobes	2	0.08
*Enterobacterales*	767	32.03
*E. coli*	270	11.27
*K. pneumoniae*	170	7.10
*Enterobacter* spp.	139	5.80
*Salmonella* spp.	28	1.17
*P. aeruginosa*	132	5.51
*Acinetobacter* spp.	75	3.13
*S. maltophilia*	22	0.92
*Achromobacter* spp.	7	0.29
*Burkholderia* spp.	4	0.17
Other non-fermenting Gram-negative	69	2.88
*N. meningitidis*	7	0.29
*H. influenzae*	13	0.54
*Brucella* spp.	1	0.04
Other Gram-negative	36	1.50
Gram-negative anaerobes	1	0.04
Total bacterial BSIs	2207	92.15
*C. albicans*	71	2.96
*C. parapsilosis*	59	2.46
*C. glabrata*	9	0.38
*C. krusei*	3	0.13
*Candida* spp.	3	0.13
Other non-albicans *Candida*	26	1.09
Other non-identified yeast	7	0.29
Other non-identified yeast/mold	10	0.42
Total fungal BSIs	188	7.85

**Table 2:** Absolute numbers and proportions of antibiotic-resistant organisms.

Pathogen	Absolute Number, *n*	Percentage of Total BSI, %
Methicillin-resistant *S. aureus* (MRSA)	97	4.05
Vancomycin-resistant *Enterococci* (VRE)	12	0.50
ESBL-producing *Enterobacterales*	94	3.92
AmpC-producing *Enterobacterales*	67	2.80
Meropenem-resistant *Enterobacterales*	10	0.42
Cefepime/ceftazidime-resistant *P. aeruginosa*	21	0.88
Piperacillin/tazobactam-resistant *P. aeruginosa*	17	0.71
Meropenem-resistant *P. aeruginosa*	6	0.25

**Table 3:** Absolute numbers and proportions of pathogens isolated from bloodstream infections, stratified by age subgroups.

Microorganism	Age ≤ 7 Days	8–30 Days	1–3 Months	3 Months–1 Year	1–5 Years	>5 Years
*S. aureus*, *n* (%)	28 (27.72)	65 (22.34)	69 (21.10)	38 (19.49)	153 (19.20)	126 (18.42)
MRSA, *n* (%)	10 (9.90)	19 (6.53)	14 (4.28)	6 (3.08)	29 (3.64)	19 (2.78)
*S. pyogenes*, *n* (%)	0 (0.00)	0 (0.00)	1 (0.31)	1 (0.51)	8 (1.00)	10 (1.46)
*S. pneumoniae*, *n* (%)	1 (0.99)	0 (0.00)	2 (0.61)	0 (0.00)	25 (3.14)	10 (1.46)
*S. agalactiae*, *n* (%)	13 (12.87)	20 (6.87)	27 (8.26)	4 (2.05)	0 (0.00)	1 (0.15)
Viridans *Streptococcus* spp., *n* (%)	0 (0)	0 (0)	1 (0.31)	5 (2.56)	75 (9.41)	59 (8.63)
*Enterococcus* spp., *n* (%)	10 (9.90)	43 (14.78)	79 (24.16)	42 (21.54)	87 (10.92)	49 (7.16)
*E. faecalis*, *n* (%)	8 (7.92)	37 (12.71)	59 (18.04)	31 (15.90)	53 (6.65)	26 (3.80)
*E. faecium*, *n* (%)	1 (0.99)	4 (1.37)	16 (4.89)	10 (5.13)	27 (3.39)	17 (2.49)
VRE, *n* (%)	0 (0.00)	1 (0.34)	0 (0.00)	2 (1.03)	7 (0.88)	2 (0.29)
*Listeria*, *n* (%)	4 (3.96)	0 (0.00)	0 (0.00)	0 (0.00)	4 (0.50)	0 (0.00)
*Enterobacterales*, *n* (%)	30 (29.70)	118 (40.55)	116 (35.47)	79 (40.51)	226 (28.36)	198 (28.95)
*E. coli*, *n* (%)	20 (19.80)	37 (12.71)	27 (8.26)	18 (.23)	61 (7.65)	107 (15.64)
*K. pneumoniae*, *n* (%)	3 (2.97)	28 (9.62)	36 (11.01)	21 (10.77)	51 (6.40)	31 (4.53)
*Enterobacter* spp., *n* (%)	2 (1.98)	14 (4.81)	27 (8.26)	20 (10.26)	50 (6.27)	26 (3.80)
ESBL producing, *n* (%)	1 (0.99)	7 (2.41)	19 (5.81)	8 (4.10)	32 (4.02)	27 (3.95)
AmpC producing, *n* (%)	1 (0.99)	6 (2.06)	11 (3.36)	10 (5.13)	27 (3.39)	12 (1.75)
Meropenem resistant, *n* (%)	0 (0.00)	0 (0.00)	0 (0.00)	0 (0.00)	6 (0.75)	4 (0.58)
*P. aeruginosa*, *n* (%)	2 (1.98)	4 (1.37)	5 (1.53)	8 (4.10)	51 (6.40)	62 (9.06)
Cefepime/ceftazidime-resistant, *n* (%)	0 (0.00)	2 (0.69)	0 (0.00)	3 (1.54)	9 (1.13)	7 (1.02)
Piperacillin/tazobactam-resistant, *n* (%)	0 (0.00)	1 (0.34)	0 (0.00)	2 (1.03)	9 (1.13)	5 (0.73)
Meropenem-resistant, *n* (%)	0 (0.00)	0 (0.00)	0 (0.00)	1 (0.51)	3 (0.38)	2 (0.29)
*N. meningitidis*, *n* (%)	0 (0.00)	0 (0.00)	0 (0.00)	1 (0.51)	2 (0.25)	4 (0.58)
*H. influenzae*, *n* (%)	1 (0.99)	0 (0.00)	1 (0.31)	1 (0.51)	8 (1.00)	2 (0.29)
*C. albicans*, *n* (%)	7 (6.93)	19 (6.53)	6 (1.83)	4 (2.05)	14 (1.76)	21 (3.07)
*C. parapsilosis*, *n* (%)	1 (0.99)	6 (2.06)	3 (0.92)	4 (2.05)	23 (2.89)	22 (3.22)
Total (bacterial + fungal) BSIs, *n* (%)	101 (100)	291 (100)	327 (100)	195 (100)	797 (100)	684 (100)

Legend: MRSA, methicillin-resistant *Staphylococcus aureus*; VRE, vancomycin-resistant *Enterococcus*; ESBL, extended-spectrum *β*-lactamases; BSIs, bloodstream infections.

**Table 4:** Absolute numbers and proportions of pathogens isolated from bloodstream infections classified as community- or hospital-onset.

Microorganism	Emergency Ward	Other Hospital Wards
*S. aureus*, *n* (%)	68 (25.76)	411 (19.29)
MRSA, *n* (%)	6 (2.27)	91 (4.27)
*S. pyogenes*, *n* (%)	12 (4.55)	8 (0.38)
*S. pneumoniae*, *n* (%)	26 (9.85)	12 (0.56)
*S. agalactiae*, *n* (%)	25 (9.47)	40 (1.88)
Viridans *Streptococcus* spp., *n* (%)	0 (0)	140 (6.6)
*Enterococcus* spp., *n* (%)	8 (3.03)	302 (14.17)
*E. faecalis*, *n* (%)	5 (1.89)	209 (9.81)
*E. faecium*, *n* (%)	3 (1.14)	72 (3.38)
VRE, *n* (%)	0 (0.00)	12 (0.56)
*Listeria*, *n* (%)	1 (0.38)	7 (0.33)
*Enterobacterales*, *n* (%)	79 (29.92)	688 (32.29)
*E. coli*, *n* (%)	46 (17.42)	224 (10.51)
*K. pneumoniae*, *n* (%)	6 (2.27)	164 (7.70)
*Enterobacter* spp., *n* (%)	7 (2.65)	132 (6.19)
ESBL-producing, *n* (%)	2 (0.76)	92 (4.32)
AmpC-producing, *n* (%)	2 (0.76)	65 (3.05)
Meropenem-resistant, *n* (%)	0 (0.00)	10 (0.47)
*P. aeruginosa*	7 (2.65)	125 (5.87)
Cefepime/ceftazidime-resistant, *n* (%)	0 (0.00)	21 (0.99)
Piperacillin/tazobactam-resistant, *n* (%)	0 (0.00)	17 (0.80)
Meropenem-resistant, *n* (%)	0 (0.00)	6 (0.28)
*N. meningitidis*, *n* (%)	5 (1.89)	2 (0.09)
*H. influenzae*, *n* (%)	4 (1.52)	9 (0.42)
*C. albicans*, *n* (%)	1 (0.38)	70 (3.28)
*C. parapsilosis*, *n* (%)	3 (1.14)	56 (2.63)
Total (bacterial + fungal) BSIs, *n* (%)	264 (100.00)	2131 (100.00)

Legend: MRSA, methicillin-resistant *Staphylococcus aureus*; VRE, vancomycin-resistant *Enterococcus*; ESBL, extended-spectrum *β*-lactamases; BSIs, bloodstream infections.

**Table 5:** Absolute number s and incidence rates per 10,000 admissions of pathogens isolated from bloodstream infections from 2006 to 2022.

Microorganism Causing bsi: Absolute Number (*n*); Incidence Rate 10,000 Hospital Admission (IR)		2006	2007	2008	2009	2010	2011	2012	2013	2014	2015	2016	2017	2018	2019	2020	2021	2022	Spearman Test	
																			r_s_	*p*-Value
All bacteria	*n* IR	90 49.43	101 58.85	107 64.92	96 56.59	100 58.57	104 60.30	114 69.61	121 82.04	106 73.35	159 116.02	149 106.87	160 109.34	175 123.64	137 92.94	149 118.30	163 123.27	176 126.59	0.85454	0.00081
*S. aureus*	*n* IR	16 8.79	18 10.49	19 11.53	15 8.90	17 9.96	15 8.70	24 14.65	27 18.31	25 17.30	27 19.70	41 29.40	28 19.13	39 27.55	41 27.81	43 34.14	44 33.28	40 28.77	0.89706	0
MRSA	*n* IR	3 1.65	4 2.33	3 1.82	3 1.78	2 1.17	2 1.16	5 3.05	2 1.36	6 4.15	9 6.57	4 2.87	5 3.42	15 10.60	9 6.11	14 11.12	6 4.54	5 3.60	0.7402	0.00068
*S. pyogenes*	*n* IR	2 1.10	4 2.33	1 0.61	1 0.59	2 1.17	0 0.00	1 0.61	0 0.00	0 0.00	2 1.46	1 0.72	0 0.00	1 0.71	3 2.04	0 0.00	1 0.76	1 0.72	−0.08448	0.74718
*S. pneumoniae*	*n* IR	3 1.65	3 1.75	6 3.64	4 2.37	6 3.51	5 2.90	0 0.00	0 0.00	0 0.00	1 0.73	2 1.43	5 3.42	1 0.71	0 0.00	0 0.00	0 0.00	2 1.44	−0.53488	0.02695
*S. agalactiae*	*n* IR	1 0.55	1 0.58	1 0.61	5 2.97	3 1.76	0 0.00	3 1.83	7 4.75	4 2.77	5 3.65	9 6.45	2 1.37	7 4.95	3 2.04	4 3.18	6 4.54	4 2.88	0.59804	0.01122
Viridans *Streptococcus* spp.	*n* IR	12 6.59	7 4.08	11 6.67	9 5.34	14 8.20	8 4.64	5 3.05	3 2.03	6 4.15	11 8.03	10 7.17	8 5.47	4 2.83	6 4.07	2 1.59	11 8.32	13 9.35	−0.18573	0.4754
*Enterococcus* spp.	*n* *IR*	9 4.94	15 8.74	12 7.28	9 5.34	11 6.44	17 9.86	22 13.43	16 10.85	15 10.38	32 23.35	18 12.91	25 17.08	32 22.61	15 10.18	16 12.70	17 12.86	29 20.86	0.72794	0.00092
*E. faecalis*	*n* *IR*	2 1.10	8 4.66	5 3.03	3 1.78	7 4.10	10 5.80	16 9.77	11 7.46	13 9.00	22 16.05	13 9.32	16 10.93	26 18.37	13 8.82	13 10.32	14 10.59	22 15.82	0.82108	<0.00005
*E. faecium*	*n* *IR*	3 1.65	5 2.91	5 3.03	5 2.97	3 1.76	5 2.90	4 2.44	4 2.71	1 0.69	9 6.57	5 3.59	9 6.15	6 4.24	1 0.68	3 2.38	3 2.27	4 2.88	−0.01471	0.95533
VRE	*n* IR	0 0.00	0 0.00	3 1.82	0 0.00	0 0.00	0 0.00	1 0.61	0 0.00	0 0.00	1 0.73	0 0.00	3 2.05	0 0.00	1 0.68	0 0.00	1 0.76	2 1.44	0.37319	0.1401
*Listeria* sp.	*n* IR	1 0.55	1 0.58	0 0.00	1 0.59	2 1.17	0 0.00	0 0.00	0 0.00	2 1.38	0 0.00	0 0.00	0 0.00	0 0.00	1 0.68	0 0.00	0 0.00	0 0.00	−0.37856	0.13403
*Enterobacterales*	*n* IR	29 15.93	28 16.32	32 19.42	35 20.76	29 16.99	41 23.77	44 26.87	47 31.87	43 29.75	53 38.67	47 33.71	59 40.32	68 48.04	49 33.24	51 40.49	57 43.11	55 39.56	0.92157	0
* E. coli*	*n* IR	11 6.04	9 5.24	17 10.31	10 5.93	17 9.96	15 8.70	18 10.99	18 12.20	11 7.61	16 11.67	15 10.76	23 15.72	25 17.66	16 10.85	11 8.73	18 13.61	20 14.39	0.69363	0.00201
* K. pneumoniae*	*n* IR	5 2.75	2 1.17	6 3.64	6 3.56	4 2.34	13 7.54	13 7.94	14 9.49	7 4.84	9 6.57	10 7.17	16 10.93	18 12.72	10 6.78	9 7.15	18 13.61	10 7.19	0.66667	0.00347
*Enterobacter* spp.	*n* IR	8 4.39	11 6.41	4 2.43	6 3.56	5 2.93	6 3.48	4 2.44	4 2.71	12 8.30	10 7.30	11 7.89	6 4.10	12 8.48	8 5.43	11 8.73	8 6.05	13 9.35	0.6152	0.00858
ESBL-producing	*n* IR	2 1.10	5 2.91	4 2.43	3 1.78	1 0.59	7 4.06	6 3.66	3 2.03	6 4.15	7 5.11	5 3.59	10 6.83	12 8.48	5 3.39	2 1.59	9 6.81	7 5.03	0.56863	0.01723
AmpC-producing	*n* IR	3 1.65	2 1.17	2 1.21	1 0.59	2 1.17	3 1.74	5 3.05	2 1.36	6 4.15	6 4.38	4 2.87	4 2.73	10 7.07	4 2.71	5 3.97	3 2.27	5 3.60	0.6634	0.00369
Meropenem-resistant	*n* IR	0 0.00	0 0.00	1 0.61	0 0.00	0 0.00	0 0.00	0 0.00	1 0.68	0 0.00	2 1.46	4 2.87	0 0.00	2 1.41	0 0.00	0 0.00	0 0.00	0 0.00	0.05474	0.83471
*P. aeruginosa*	*n* IR	5 2.75	7 4.08	9 5.46	9 5.34	7 4.10	7 4.06	7 4.27	7 4.75	5 3.46	12 8.76	7 5.02	11 7.52	10 7.07	4 2.71	7 5.56	8 6.05	10 7.19	0.4951	0.04331
Cefepime/ceftazidime-resistant	*n* IR	1 0.55	2 1.17	0 0.00	1 0.59	2 1.17	0 0.00	0 0.00	2 1.36	0 0.00	6 4.38	0 0.00	2 1.37	2 1.41	0 0.00	1 0.79	0 0.00	2 1.44	0.17916	0.49142
Piperacillin/tazobactam-resistant	*n* IR	1 0.55	0 0.00	0 0.00	0 0.00	1 0.59	0 0.00	0 0.00	2 1.36	0 0.00	4 2.92	1 0.72	2 1.37	3 2.12	0 0.00	1 0.79	0 0.00	2 1.44	0.42052	0.09281
Meropenem-resistant	*n* IR	0 0.00	0 0.00	0 0.00	0 0.00	0 0.00	0 0.00	0 0.00	0 0.00	0 0.00	3 2.19	0 0.00	0 0.00	2 1.41	0 0.00	1 0.79	0 0.00	0 0.00	0.32612	0.20144
*N. meningitidis*	*n* IR	0 0.00	1 0.58	0 0.00	0 0.00	2 1.17	0 0.00	0 0.00	0 0.00	1 0.69	0 0.00	0 0.00	1 0.68	0 0.00	1 0.68	1 0.79	0 0.00	0 0.00	0.09759	0.70943
*H. influenzae*	*n* IR	0 0.00	0 0.00	2 1.21	1 0.59	0 0.00	0 0.00	1 0.61	0 0.00	0 0.00	0 0.00	1 0.72	3 2.05	1 0.71	0 0.00	0 0.00	2 1.51	2 1.44	0.38747	0.12438
All fungal spp.	*n* IR	13 7.14	8 4.66	11 6.67	20 11.86	11 6.44	12 6.96	12 7.33	9 6.10	12 8.30	14 10.22	17 12.19	11 7.52	9 6.36	5 3.39	9 7.15	3 2.27	12 8.63	0.01226	0.96277
*C. albicans*	*n* IR	4 2.20	1 0.58	4 2.43	9 5.34	4 2.34	8 4.64	4 2.44	5 3.39	8 5.54	4 2.92	3 2.15	4 2.73	3 2.12	1 0.68	3 2.38	1 0.76	5 3.60	−0.08088	0.75763
*C. parapsilosis*	*n* IR	5 2.75	3 1.75	6 3.64	5 2.97	2 1.17	3 1.83	3 1.74	1 0.68	3 2.08	6 4.38	7 5.02	3 2.05	3 2.12	2 1.36	4 3.18	0 0.00	3 2.16	−0.05637	0.82985

Legend: MRSA, methicillin-resistant *Staphylococcus aureus*; VRE, vancomycin-resistant *Enterococcus*; ESBL, extended-spectrum *β*-lactamases; BSIs, bloodstream infections.

## Data Availability

The data are contained within the article. They are available upon request from the corresponding author.

## Supplementary Materials

The following supporting information can be downloaded at: https://www.mdpi.com/article/10.3390/pathogens13080675/s1. Table S1. Blood culture’s contaminant microorganism. References [14,15,16,17] are cited also in Supplementary Materials.
