# Supplementary material for: The Etiology of Bloodstream Infections at an Italian Pediatric Tertiary Care Hospital: A 17-Year-Long Series
Source: Pathogens. 2024 Aug 9;13(8):675. doi: 10.3390/pathogens13080675 (PMC11357311; doi:10.3390/pathogens13080675)
Supplement: Supplementary file 1 [file pathogens-13-00675-s001.zip › pathogens-3085953-supplementary.pdf]

Supplementary Table S1. Blood culture's contaminant microorganism.

**Microorganisms**	**Contamination rate (%)**
**Gram-positive bacteria**	
Coagulase-negative *Staphylococci*	62-63 [1], 81.9 [2]
Corynebacterium spp. (other than C. jeikieum)	68-78 [1], 96.2 [2]
Bacillus spp	68-70 (other than *B. anthracis*) [1], 91.7 [2]
**Anaerobic bacteria**	
Propionibacterium acnes	84-85 [1], 100 [2]
Clostridiium perfringens	Not reported [1], 76.9 [2]

**S. epidermidis, S. haemolyticus, S. capitis, S. hominis, S. pettenkoferi, S. simulans, S. warneri, S. saprophytocus* and others. Animal associated and other CoNS: *S. carnosus, S. caprae, S. lentus* and others[5]. Despite *S. lugdunensis* belong to the CoNS, we did not consider it as a contaminant.

***Micrococcus* spp. [3]

REFERENCES

- Weinstein, M.P.; Towns, M.L.; Quartey, S.M.; Mirrett, S.; Reimer, L.G.; Parmigiani, G.; Reller, L.B. The clinical significance of positive blood cultures in the 1990s: A prospective comprehensive evaluation of the microbiology, epidemiology, and outcome of bacteremia and fungemia in adults. Clin. Infect. Dis. 1997, 24, 584–602. https://doi.org/10.1093/clind/24.4.584.
- Hall, K.K.; Lyman, J.A. Updated review of blood culture contamination. Clin. Microbiol. Rev. 2006, 19, 788–802. https://doi.org/10.1128/CMR.00062-05.
- Schifman, R.B.; Strand, C.L.; Meier, F.A.; Howanitz, P.J. Blood culture contamination: A College of American Pathologists Q-Probes study involving 640 institutions and 497134 specimens from adult patients. Arch. Pathol. Lab. Med. 1998, 122, 216–221.
- Becker K, Both A, Weißelberg S, Heilmann C, Rohde H. Emergence of coagulase-negative staphylococci. Expert Rev Anti Infect Ther, 2020. 18(4): p. 349-366.
